# Revelation of the discrepancy of volatile compounds in fig (*Ficus carica*) via gas chromatography ion‐mobility spectrometry

**DOI:** 10.1002/fsn3.3843

**Published:** 2024-01-24

**Authors:** Xinyu Liu, Rui Sun, Qiu Wu, Ming Jia, Tingjuan Yu, Yanling Han, Mingguan Yang, Lei Sun

**Affiliations:** ^1^ School of Food Science and Engineering Qilu University of Technology (Shandong Academy of Sciences) Jinan Shandong China; ^2^ College of Life Sciences Shandong Normal University Jinan China; ^3^ Economic Forest Institute Shandong Academy of Forestry Sciences Jian Shandong China; ^4^ Jian Forestry and Fruit Technology Promotion and Industrial Service Center Jinan Shandong China

**Keywords:** figs, gas chromatography ion mobility spectrometry, principal component analysis, volatile compounds

## Abstract

The volatile compounds of fig *(Ficus carica)* are influenced by various factors. To explore the composition and difference of volatile compounds among figs, gas chromatography ion mobility spectrometry (GC‐IMS) was used to study the volatiles of figs from various regions, diverse cultivars, and after treatment with different drying methods. Aldehydes were the main volatile compounds in Bojihong from Shandong, while esters, ketones, and alcohols were the main volatile compounds in Bojihong from Sichuan and Guangdong. The volatiles of Branswick and Banane were similar, but differed significantly from those of Bojihong. Drying had the most significant effect on fig volatiles, which greatly reduced the content of benzaldehyde, (E)‐2‐hexenal, 2‐methylbutanal aldehydes, lost the content of esters such as isoamyl acetate, butyl acetate, ethyl butyrate, and generated some ketones and ethers. The results showed that Bojihong from Shandong was more suitable for the processing of subsequent fig drying products.

## Graphical abstract

In this paper, we analyzed the volatile compound in fig by GC‐IMS combined with PCA method, and concluded that Bojihong from Shandong was more suitable for subsequent drying and processing of figs.

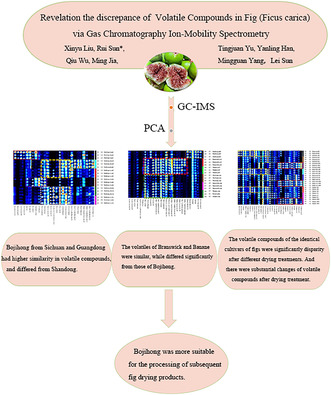

## 1 INTRODUCTION

Fig (*Ficus carica* Linn), belonging to the genus *Ficus* of the Moraceae family, is one of the most widely grown berries in the world (Mirheidari et al., 2020). Figs are extremely nutrient‐rich and exert anti‐cancer and antioxidant effects (Adiletta et al., 2019; Essa et al., 2015). Their unique fragrance is also loved by people and is mainly eaten fresh in China. Most studies on figs have focused on the effects of biotic (Abdolinejad et al., 2021; Ammar et al., 2022) and abiotic (Arvaniti et al., 2019; Backes et al., 2018; Gharibzahedi et al., 2019; Yu et al., 2020) stresses, and only a few studies have investigated the variation and variance analysis of the volatile organic compounds (VOCS) of fig. For previous works, GC–MS was the primary method for detecting volatile organic compounds in fruit, including the analysis and comparison of VOCS such as oranges, durians, pomegranates, and blueberries (Beaulieu & Stein‐Chisholm, 2016; Cuevas et al., 2017; Pico et al., 2022; Xiao et al., 2022).

Volatile compounds are one of the most important indicators of fruit quality (Cagliero et al., 2012). The unique volatile compounds in figs are influenced by various aspects (Villalobos et al., 2018), such as cultivars, geographical location, and processing methods. In research conducted by Fella et al., the composition of VOCS in ripe fruit changed, and Zidi et al. stated that the VOCS of fig fruit had different characteristics in distinct ripening stages (Fella et al., 2022; Zidi et al., 2021). Figs are widely grown in China, and the main cultivars are Bojihong, Branswick, and Banane. Figs from different regions and cultivars have distinct aromas and contain various volatile components. Therefore, it is important to study the volatile components of figs from various regions and cultivars for the subsequent selection and breeding of seeds, consumption of fresh fruits, and processing of products.

As figs are perishable and difficult to store (Paolucci et al., 2020), drying has become an important way to preserve figs in China (Slatnar et al., 2011). The most common drying treatments are natural drying, vacuum drying, hot air drying, microwave drying, freeze drying, and explosion puffing drying. Hot air drying and freeze drying are the most common drying methods in fruit and vegetable processing. Vallejo et al. (2012)) found that dried figs had higher total amounts of phenolics than fresh fruits. Explosion puffing drying can make the product taste crispy and retain the nutrients of the dried product, and is mostly used in combination with other drying techniques (Chen et al., 2017). As one of the main processing methods for figs, drying has different effects on the volatile substances of figs during processing.

Gas chromatography–mass spectrometry (GC–MS) has been used to identify the volatile compounds in figs (Mujić et al., 2012). Although it can be easily operated using pretreatment methods such as HiSorb, it still has the disadvantages of being time‐consuming, high cost, the complexity of food matrices, and the isobars and isomers that cannot be separated by MS (Fella et al., 2023; Hearn et al., 2022; Hernández‐Mesa et al., 2019). By contrast, ion mobility chromatography (IMS) is an ultra‐sensitive, efficient, and easy‐to‐use technique for the detection of trace gases. The combination of GC and IMS allows not only analysis at normal pressure but also differential analysis and the generation of visual fingerprints, thereby making data processing easier (Castell et al., 2022; Leng et al., 2021; Liu et al., 2021). However, there are only a few studies on the application of gas phase–ion mobility chromatography (GC‐IMS) to analyze the combined volatile compound composition and differentiation of fresh and dried figs.

To our knowledge, there is no reporting comparing the VOCS of the above‐mentioned regions, cultivars, and after drying treatment of figs. In this study, the volatile compounds of figs from different regions and cultivars and those produced using different drying treatments were comparatively analyzed using GC‐IMS, fingerprinting, and principal component analysis (PCA). Our findings lay the theoretical foundation for the selection and breeding of new figs in China, the consumption of fresh fruits, and the processing of products.

## 2 MATERIALS AND METHODS

### 2.1 Sample collection and preparation

**FIGURE 1:**
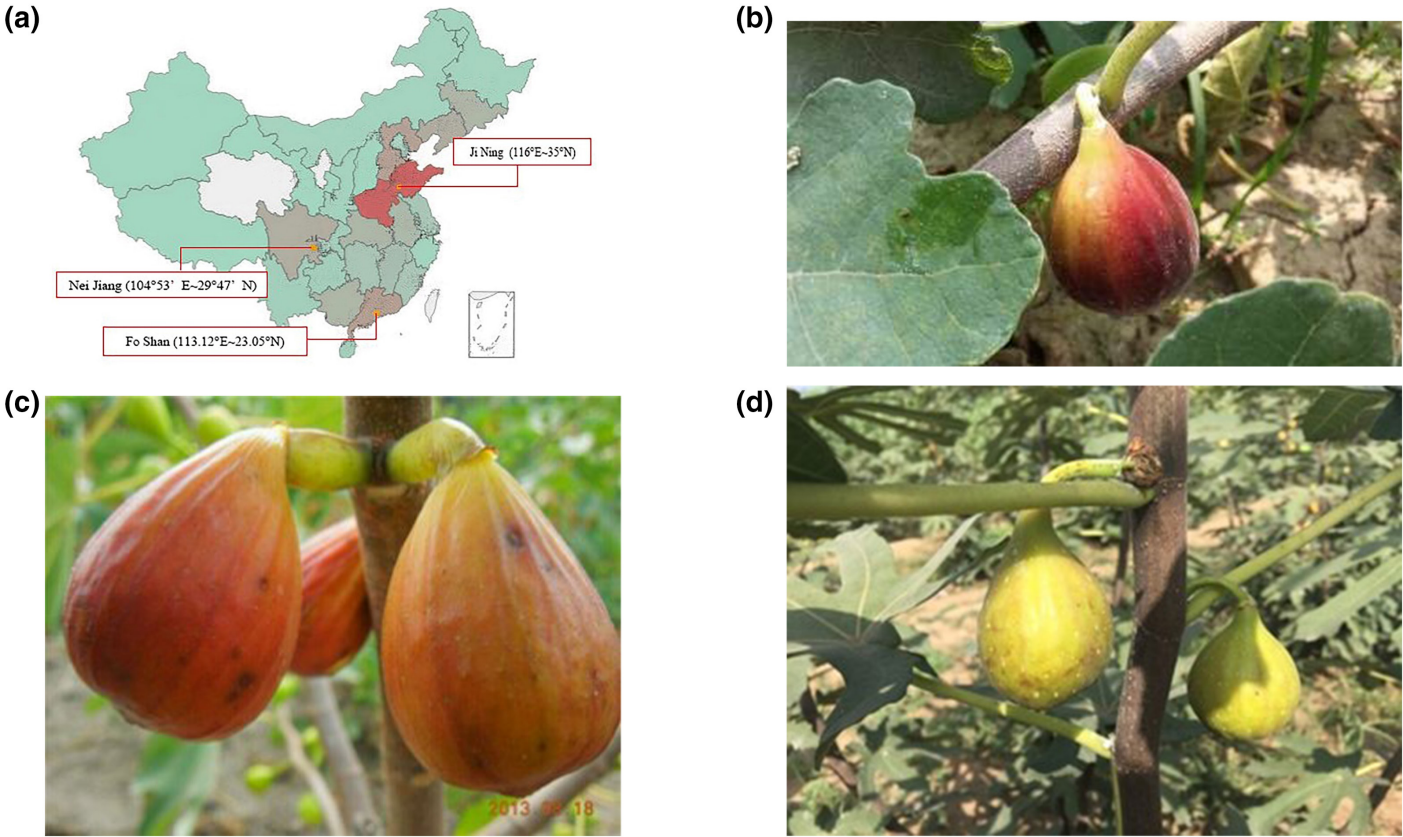
Pictures of figs from different regions and cultivars. (a) Schematic diagram of the fig sampling areas in China. (b) Bojihong; (c) Branswick; (d) Banane cultivars.

To investigate differences in the volatile compounds of figs from different regions, samples were collected from Jiaxiang County, Jining City, Shandong Province, China (116° E, 35° N); Weiyuan County, Neijiang City, Sichuan Province (104°16′–104°53′ E, 29°22′–29°47′ N); and Foshan City, Guangdong Province (113.12° E, 23.05° N), as shown in Figure 1a. All figs were collected at maturity stage and named Bojihong‐sd‐peel, Bojihong‐sd‐pulp, Bojihong‐gd‐peel, Bojihong‐gd‐pulp, Bojihong‐sc‐peel, and Bojihong‐sc‐pulp.

To study differences in the volatile compounds of figs among cultivars, samples of three cultivars (Bojihong, Branswick, and Banane) were collected from Shandong Province, China (Figure 1b–d).

To study differences in volatile compounds among figs after different drying treatments (hot air drying (HD), freeze‐drying (FD), and explosion puffing drying (EPD)), the above three figs were treated with three drying methods and were named as Bojihong‐HD, Bojihong‐FD, Bojihong‐EPD, Branswick‐HD, Branswick‐FD, Branswick‐EPD, Banane‐HD, Banane‐FD, and Banane‐EPD.

### 2.2 Dry pretreatment

#### 2.2.1 Hot air drying (HD)

Fresh figs were sliced to a thickness of 5 mm and dried for 24 h at a regulated air speed of 0.5 m/s and a temperature of 60°C. The moisture content of the dried figs was less than 10%.

#### 2.2.2 Freeze‐drying (FD)

Fresh fruits were sliced to a thickness of 5 mm, prefrozen at −80°C for 12 h, and then freeze‐dried in a vacuum at 1 kPa and a tray temperature of 27°C for 48 h. The moisture content of the dried figs was less than 10%.

#### 2.2.3 Explosion puffing drying (EPD)

Fresh figs were sliced to a thickness of 1 cm and pre‐dried at 80°C for 4 h to maintain a moisture content of approximately 30%, after which they were closed and softened for 24 h. After softening, puffing was carried out at 85°C with a pressure of 0.1 MPa and a puffing time of 5 min. After 5 min, the vacuum valve was opened to allow the figs to puff rapidly, after which the temperature was cooled to 60°C, and the samples were kept in a vacuum for 2.5 h. After completion, cooling water was passed over the fruits to cool the samples to 20°C, and this temperature was maintained for 10 min, after which a normal pressure was applied. After drying, the moisture content of the figs was less than 10%.

### 2.3 GC‐IMS analysis

Volatile compounds were analyzed by GC‐IMS (FlavourSpec®, the G.A.S. Department of Shandong Haineng Science Instrument Co., Ltd., Shandong, China). The samples were weighed at 2 g into a 20‐mL headspace vial and then incubated at 500 rpm at 40°C for 15 min. 200 μL of the sample was injected into a head injector at 45°C.

GC‐IMS was carried out for 20 min using an Agilent gas chromatogram (FS‐SE‐54‐CB‐1, 15 m ID: 0.53 mm GC column) with a column temperature of 60°C and N_2_ flow rate of 150 mL/min in a capillary split column. The flow rate program was 2 mL/min for 2 min and 100 mL/min for 18 min. The NIST and IMS databases built into the software were used to identify volatile compounds.

### 2.4 Statistical analysis

Data were collected and processed using Laboratory Analytical Viewer Gallery Plot 2.2.1 software of the analytical instrument, and fingerprints were drawn by Reporter and Gallery Plot plug‐ins, while PCA was performed using SIMCA 14.1 (UMetrics AB, Umea, Sweden). The R language was used for the cluster analysis and heat map. All data were made in triplicate and presented as mean ± standard deviation.

## 3 RESULTS

### 3.1 Effect of regions on volatile compounds in figs

**FIGURE 2:**
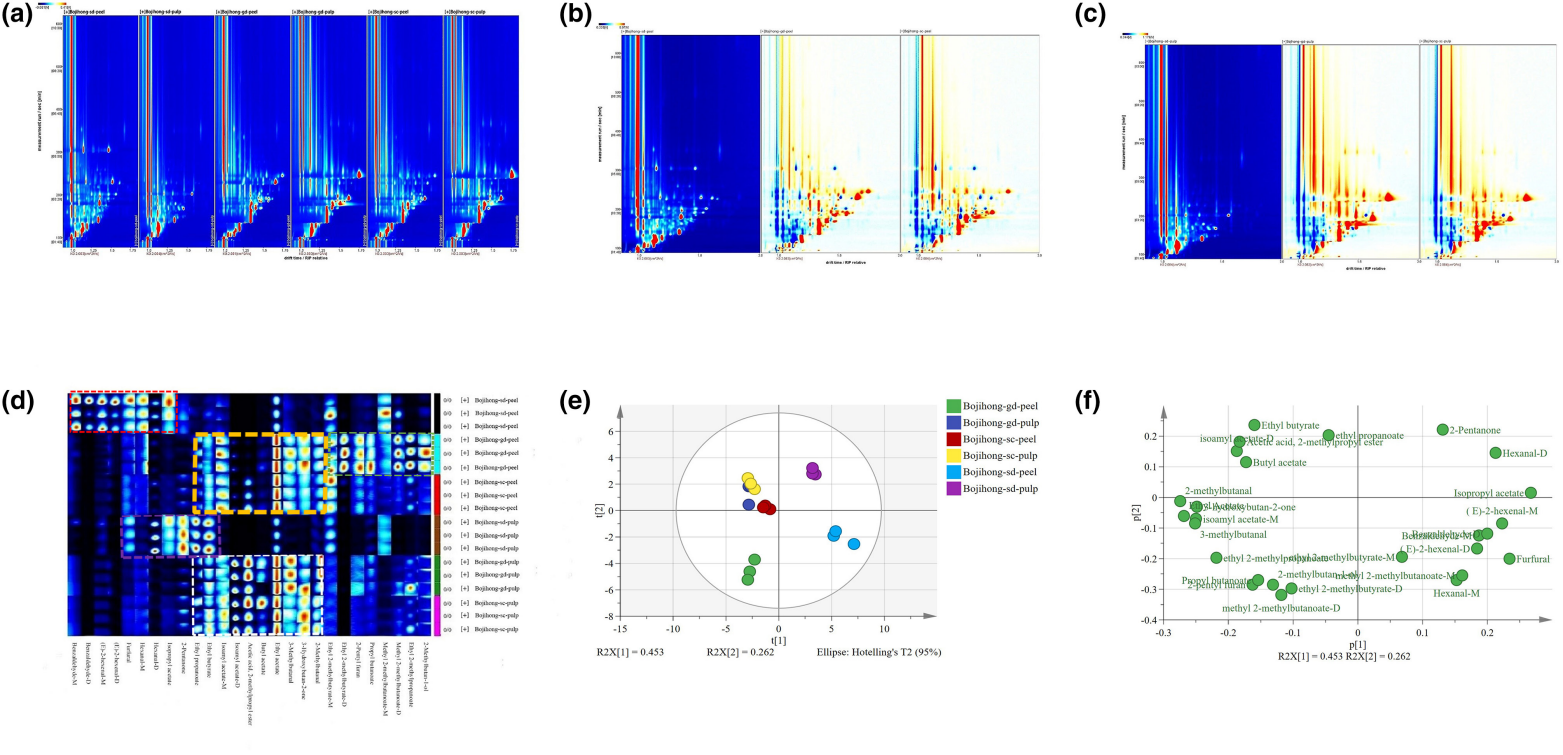
GC‐IMS analysis of three different regions of figs. (a) 2D‐topographic plots; (b and c) the difference comparison topographic plots; (d) fingerprints of volatile compounds; (e) PCA plot; and (f) loading scatter plot.

To study the difference in volatile compounds of figs caused by different regions, the GC‐IMS spectra of six fig samples (Bojihong‐sd‐peel, Bojihong‐sd‐pulp, Bojihong‐gd‐peel, Bojihong‐gd‐pulp, Bojihong‐sc‐peel, and Bojihong‐sc‐pulp) were analyzed. Figure 2a shows the 2D topographic spectra of volatile substances in fig samples from different regions that could be used for qualitative analysis. The background of GC‐IMS was blue, and the red vertical line at abscissa 1.0 was the normalized active ion peak (RIP). Each point on either side of the RIP peak represented a volatile compound, and the color reflected the concentration of the compound, with white color representing a lower concentration and red meaning a higher concentration. Most of the peak signals had a retention time of 100–600 s and a drift time of 1.0–1.75 s. In order to distinguish the differences among the fig samples from different origins more clearly, the topographical plots of other samples were derived in Figure 2b,c with Bojihong‐sd‐peel and Bojihong‐sd‐pulp as references, respectively. The red color indicates a higher concentration than the reference substance, and the blue color indicates a lower concentration than the reference substance. There were a large number of red and blue dots in Figure 2b,c, which demonstrated a significant difference among the fig samples.

GC‐IMS spectroscopy was used to identify all volatile compounds, and the Gallery Plot plug‐in was used to generate fingerprints (Figure 2d). A total of 27 volatile compounds were detected, including 14 esters, 9 aldehydes, 2 ketones, 1 alcohol, and 1 furan. Hexanal (M and D), furfural, (E)‐2‐hexenal (M and D), benzaldehyde (M and D), and isopropyl acetate were detected in Bojihong‐sd‐peel, which were significantly higher than those in other samples (labeled with a red rectangle). While ethyl butyrate, ethyl propanoate, 2‐pentanone, isopropyl acetate, hexanal (M and D), and furfural were unique in Bojihong‐sd‐pulp (labeled with purple rectangle). Figs from Sichuan and Guangdong had high similarity in volatile compounds 2‐methylbutanal, 3‐hydroxybutan‐2‐one, 3‐methylbutanal, ethyl acetate, butyl acetate, isoamyl acetate (M and D), ethyl butyrate, ethyl propanoate, and acetic acid; 2‐methylpropyl was detected in the whole fruit of Bojihong‐sc and Bojihong‐gd (labeled with white and yellow rectangles). In addition, ethyl 2‐methylbutyrate (M and D), 2‐pentyl furan, propyl butanoate, methyl 2‐methylbutanoate (M and D), ethyl 2‐methylpropanoate, and 2‐methylbutan‐1‐ol were detected as unique volatile compounds in Bojihong‐gd‐peel (labeled with a green rectangle).

Principal component analysis (PCA) was conducted to further compare the differences in volatile compounds among Bojihong figs from three regions. As shown in Figure 2e, figs from Sichuan and Guangdong were close to each other. However, the peel and pulp of figs from the same regions were separately distributed. The load chart showed that Bojihong‐sc and Bojihong‐gd were mainly esters and ketones, while Bojihong‐sd was mainly aldehydes (Figure 2f).

### 3.2 Effect of cultivars on volatile compounds in figs

**FIGURE 3:**
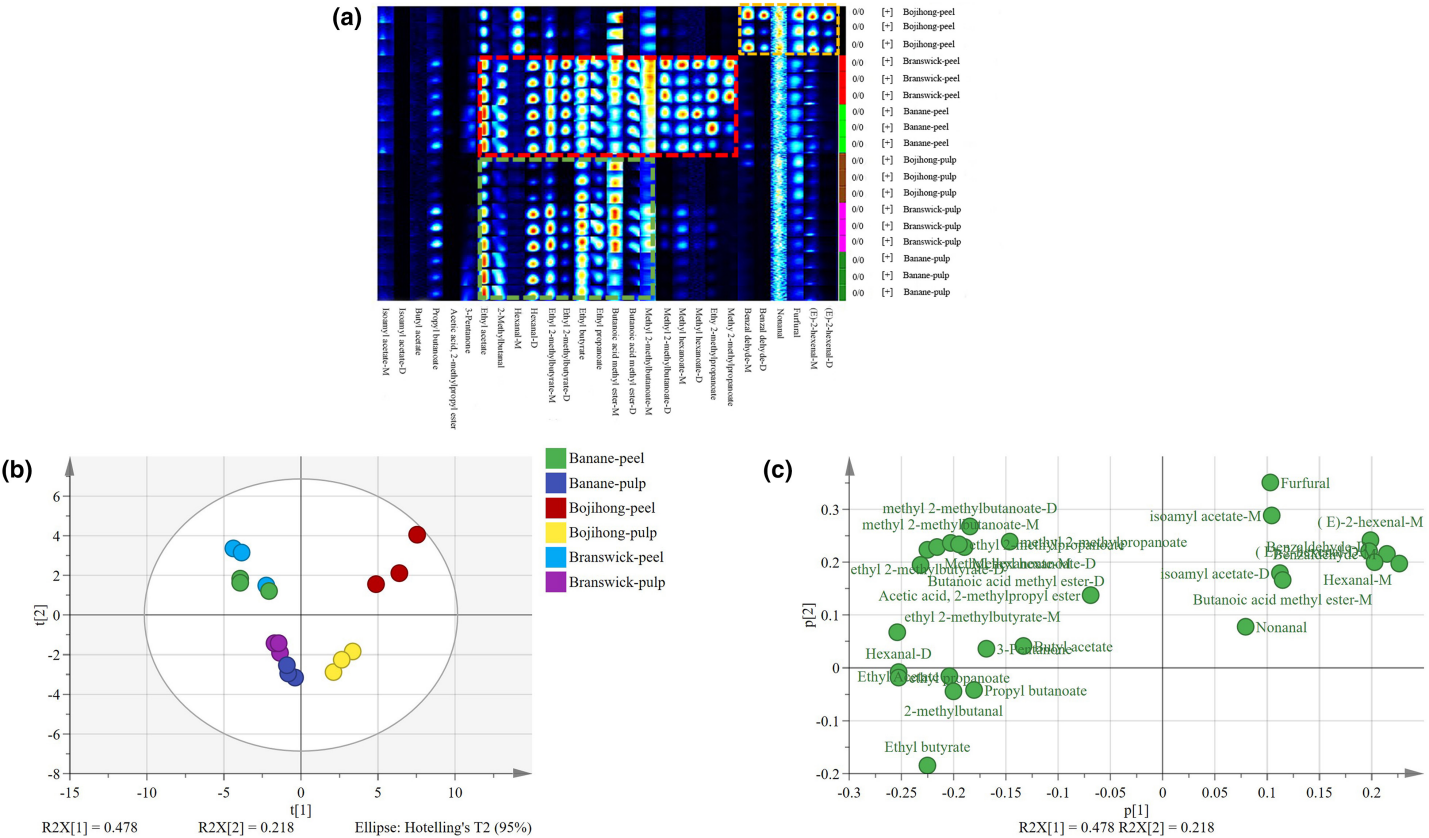
GC‐IMS analysis of three different cultivars of figs. (a) Fingerprints of volatile compounds; (b) PCA plot; and (c) loading scatter plot.

Similarly, the volatile compounds of three fig samples (Bojihong, Branswick, and Banane) were analyzed by fingerprint analysis (Figure 3). Twenty‐eight volatile compounds were detected, including 18 esters, 9 aldehydes, and 1 ketone (Figure 3a). (E)‐2‐hexenal (M and D), benzaldehyde (M and D), furfural, and nonanal were majorly present in Bojihong‐peel samples and revealed low concentrations in Branswick and Banane (labeled with a yellow rectangle). The volatile compounds, including methyl 2‐methylbutanoate‐M, butanoic acid methyl ester (M and D), ethyl propanoate, ethyl butyrate, ethyl 2‐methylbutyrate (M and D), hexanal (M and D), 2‐methylbutanal, and ethyl acetate, had been detected in all three cultivars of fig samples (labeled with a green rectangle). In addition, methyl 2‐methylpropanoate, ethyl 2‐methylpropanoate, methyl hexanoate (M and D), and methyl 2‐methylbutanoate‐D were detected in Branswick‐peel and Banane‐peel (labeled with a red rectangle).

The results of PCA in Figure 3b showed that, except for Bojihong, Branswick and Banane had similar volatile compounds. It was observed that a number of esters and ketones are in Branswick and Banane, and some aldehydes and a few esters are in Bojihong based on the load chart (Figure 3c).

### 3.3 Effect of drying treatments on volatile compounds in figs

**FIGURE 4:**
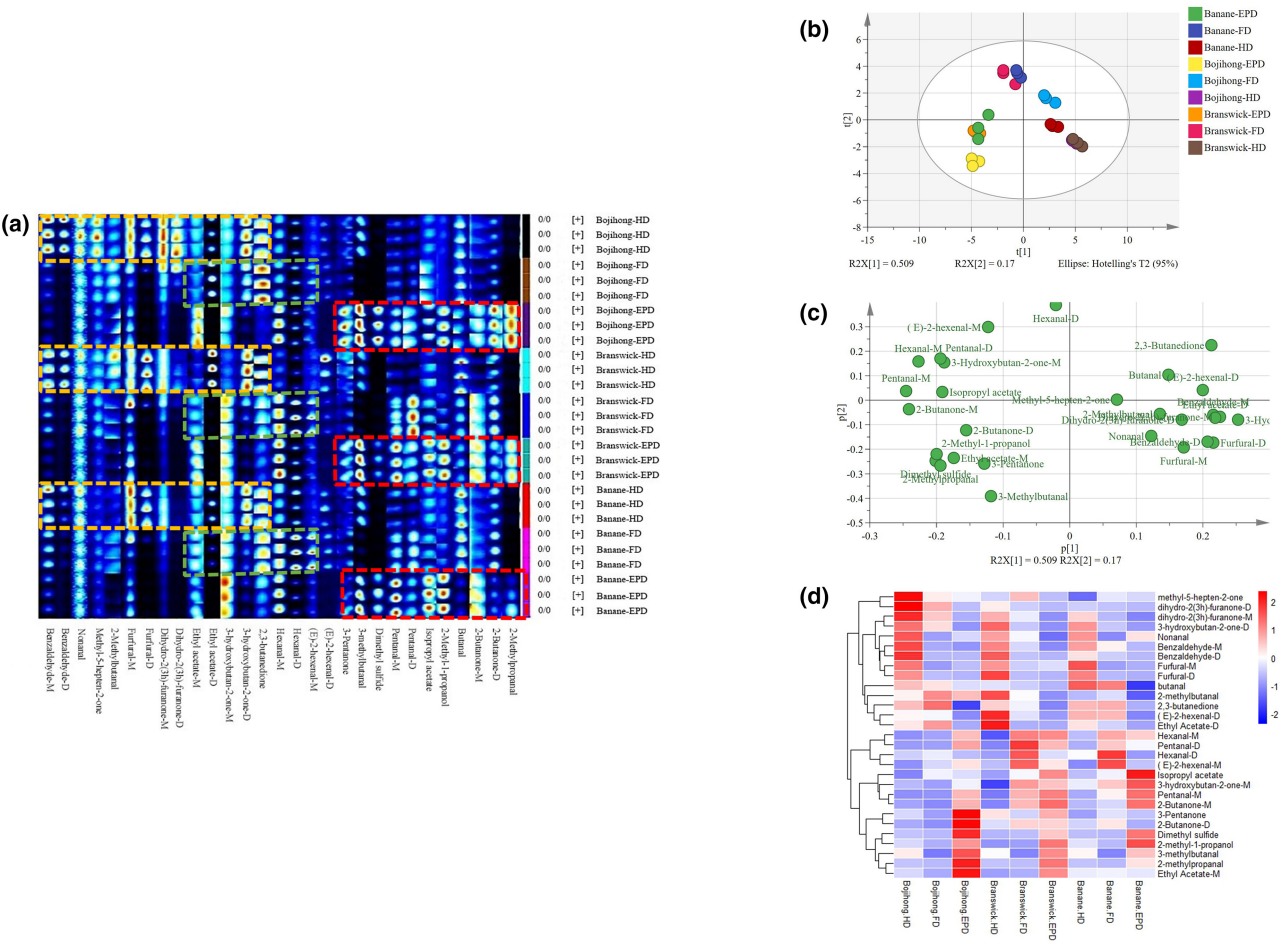
GC‐IMS analysis of three different drying treatments of figs. (a) Fingerprints of volatile compounds; (b) PCA plot; (c) loading scatter plot; and (d) heat map.

**FIGURE 5:**
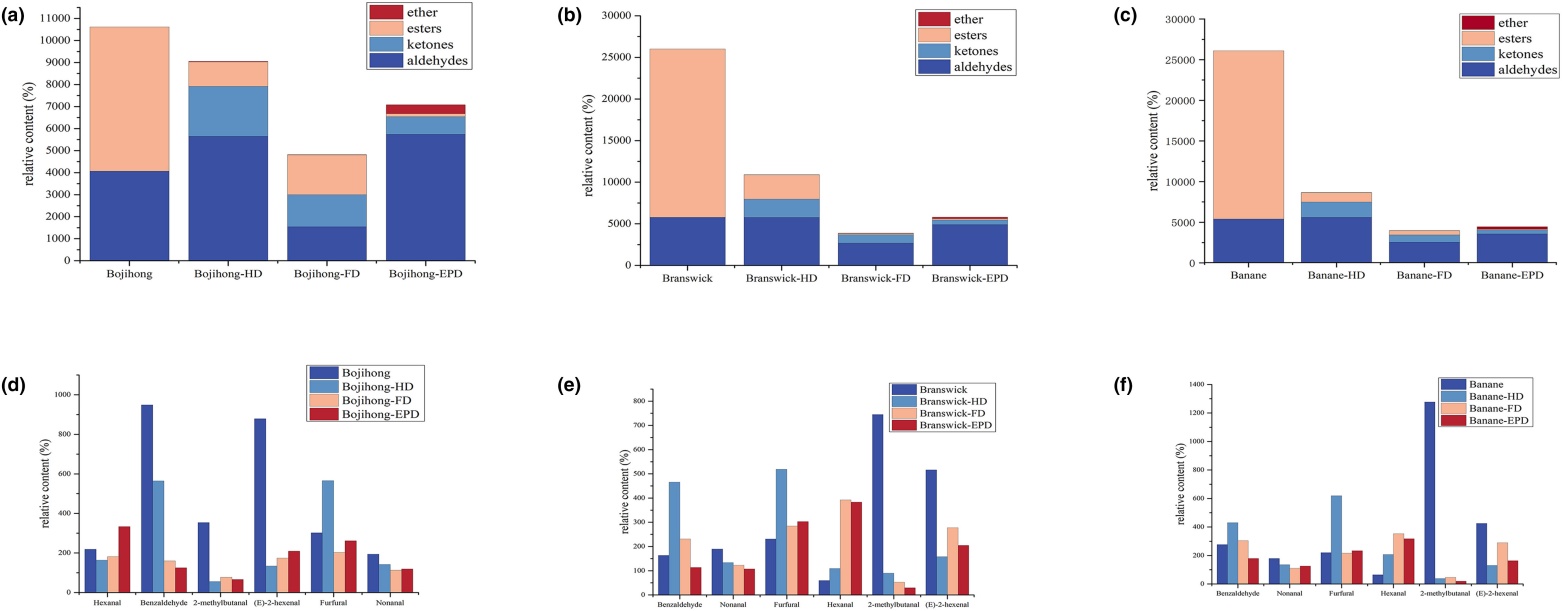
Changes in the quantity of volatile compounds of figs. (a) Changes in the quantity of volatile compounds in Bojihong after drying; (b) changes in the quantity of volatile compounds in Branswick after drying; (c) changes in the quantity of volatile compounds in Banane after drying; (d) changes in the quantity of aldehydes in Bojihong after drying; (e) changes in the quantity of aldehydes in Branswick after drying; and (f) changes in the quantity of aldehydes in Banane after drying.

Figure 4a shows the GC‐IMS fingerprints of three fig samples (Bojihong, Branswick, and Banane) treated by three drying methods (HD, FD, and EPD). The results showed that the volatiles of different cultivars of figs were related to the processing methods. There were 29 identified volatile compounds, including 15 aldehydes, 7 ketones, 3 esters, 2 furans, 1 ether, and 1 alcohol after drying. All three fig samples after EPD identified 11 compounds, including 2‐methylpropanal, 2‐butanone (M and D), butanal, 2‐methyl‐1‐propanol, isopropyl acetate, pentanal (M and D), dimethyl sulfide, 3‐methylbutanal, and 3‐pentanone, but were detected in low concentrations or could not be detected in Bojihong‐HD and Bojihong‐FD (labeled with a red rectangle). (E)‐2‐hexenal‐M, and hexanal (M and D) were specific to the three cultivars of figs treated with FD. 2,3‐Butandione, 3‐hydroxybutan‐2‐one (M and D), and ethyl acetate (M and D) were detected in high concentrations after being treated with HD and FD. In addition, dihydro‐2(3 h)‐furanone (M and D), furfural (M and D), 2‐methlbutanal, methyl‐5‐hepten‐2‐one, nonanal, and benzaldehyde (M and D) were specific to the three cultivars of figs treated with HD. The results of PCA in Figure 4b showed that the volatile compounds were significantly different after three drying methods, but the volatile compounds of different cultivars of figs after the same drying method were similar. Further difference analysis of the fig samples showed that the main compounds after HD and FD treatment were aldehydes, and the main compounds after EPD treatment were ketones, ethers, esters, and a small number of alcohols (Figure 4b,c). The volatile compounds obtained by GC‐IMS were analyzed and clustered, and the concentration of each volatile compound was indicated by different colors in the heat map (Figure 4d). The results were the same as the PCA results. Drying treatment caused a significant decrease in esters, with a contemporary increase in ketones and ethers (Figure 5a–f). Aldehydes also showed significant changes after drying treatment; the most remarkable difference was a significant decrease in benzaldehyde and (E)‐2‐hexenal in Bojihong and 2‐methylbutanal in Branswick and Banane. However, 3‐methylbutanal, butanal, and 2‐methylpropanal were generated after drying treatment. It could also be seen from the table that most of the esters of the three varieties of figs were lost after drying, and isopropyl acetate was generated (Table 1). In addition, methyl‐5‐hepten‐2‐one, dihydro‐2(3 h)‐furanone (M and D), 3‐hydroxybutan‐2‐one (M and D), 2,3‐butanedione, 2‐Butanone (M and D), dimethyl sulfide, and 2‐methyl‐1‐propanol were generated.

**TABLE 1:** Volatile compounds of figs after different drying treatments.

Compound	CAS#	Molecular formula	MW	RI	RT	DT	Drying methods	Peak volume (mean ± SD)		
								Bojihong	Branswick	Banane
Benzaldehyde‐M	C100527	C7H6O	106.100	958.900	310.545	1.152	Fresh	948.605 ± 274.831a	163.432 ± 8.459c	277.092 ± 64.664c
							HD	564.223 ± 30.553b	465.954 ± 25.788a	430.355 ± 90.466b
							FD	160.271 ± 15.264c	231.022 ± 7.163b	304.298 ± 8.945b
							EPD	125.318 ± 5.797c	113.841 ± 8.157c	179.612 ± 17.795c
Benzaldehyde‐D	C100527	C7H6O	106.100	952.000	304.831	1.475	Fresh	820.467 ± 507.626a	89.611 ± 8.954c	111.780 ± 20.706a
							HD	390.223 ± 49.864ab	344.437 ± 22.107b	185.537 ± 53.595a
							FD	62.264 ± 8.044b	76.691 ± 8.253c	91.726 ± 2.588b
							EPD	64.344 ± 5.224b	57.210 ± 7.753c	63.858 ± 3.848b
Nonanal	C124196	C9H18O	142.200	1110.500	502.450	1.473	Fresh	193.861 ± 19.476b	189.775 ± 10.097a	179.571 ± 8.148b
							HD	141.864 ± 6.524c	133.869 ± 16.170b	135.153 ± 17.275c
							FD	113.251 ± 0.880c	122.756 ± 18.438b	110.663 ± 2.427c
							EPD	119.072 ± 15.548c	107.687 ± 7.650b	126.326 ± 13.101c
Furfural	C98011	C5H4O2	96.100	832.800	224.681	1.084	Fresh	301.824 ± 50.866a	231.078 ± 14.247ab	220.250 ± 11.907b
Furfural‐M	C98011	C5H4O2	96.100	832.800	224.681	1.084	HD	565.465 ± 21.188a	518.675 ± 11.391b	619.629 ± 5.343a
							FD	202.577 ± 92.993b	284.478 ± 162.275b	215.959 ± 122.877b
							EPD	261.649 ± 25.028b	303.119 ± 54.195b	233.939 ± 43.245b
Furfural‐D	C98011	C5H4O2	96.100	830.900	223.722	1.336	HD	1793.122 ± 222.218a	2501.451 ± 116.911a	1926.145 ± 190.140a
							FD	231.846 ± 188.934b	205.309 ± 226.643b	145.527 ± 130.837b
							EPD	112.009 ± 7.059b	110.431 ± 29.630b	88.865 ± 9.027b
Hexanal‐M	C66251	C6H12O	100.200	792.700	204.197	1.257	Fresh	218.855 ± 12.495b	59.535 ± 3.494b	64.259 ± 1.282c
							HD	163.820 ± 6.613c	109.270 ± 5.633b	206.703 ± 26.433b
							FD	181.937 ± 0.103bc	392.463 ± 48.871c	353.338 ± 17.129a
							EPD	333.649 ± 27.654a	383.220 ± 46.956c	317.876 ± 19.361a
Hexanal‐D	C66251	C6H12O	100.200	792.100	203.895	1.566	Fresh	721.429 ± 74.455a	4867.286 ± 261.655a	4414.965 ± 291.049a
							HD	320.073 ± 14.695bc	221.953 ± 11.830c	412.536 ± 79.243c
							FD	384.754 ± 10.286b	776.835 ± 94.595b	834.075 ± 38.713b
							EPD	279.313 ± 23.179c	349.431 ± 87.427c	204.779 ± 26.019c
(E)‐2‐hexenal‐M	C6728263	C6H10O	98.100	847.200	232.000	1.183	Fresh	878.989 ± 142.098a	516.479 ± 24.043b	425.341 ± 51.864a
							HD	134.241 ± 13.624b	158.084 ± 7.643d	130.728 ± 8.406c
							FD	174.332 ± 19.488b	277.932 ± 38.321c	289.383 ± 15.375b
							EPD	209.507 ± 15.332b	204.892 ± 7.401d	163.434 ± 2.395c
(E)‐2‐hexenal‐D	C6728263	C6H10O	98.100	847.200	232.000	1.519	Fresh	696.716 ± 345.410a	139.049 ± 6.730a	165.426 ± 12.599a
							HD	53.139 ± 4.739b	102.790 ± 35.970a	69.405 ± 7.286b
							FD	49.406 ± 2.960b	44.472 ± 2.371b	67.499 ± 1.963b
							EPD	23.759 ± 1.816b	27.782 ± 1.339b	22.854 ± 3.018c
3‐Methylbutanal	C590863	C5H10O	86.100	662.300	157.771	1.415	Fresh	—	—	—
							HD	1581.442 ± 39.558b	1368.970 ± 147.117b	1503.974 ± 127.102b
							FD	106.614 ± 0.450c	170.847 ± 10.380c	209.839 ± 11.559c
							EPD	3152.499 ± 33.935a	2591.813 ± 117.043a	2004.921 ± 363.351b
2‐Methylbutanal	C96173	C5H10O	86.100	701.700	168.726	1.401	Fresh	353.674 ± 34.945a	744.983 ± 58.071a	1277.147 ± 167.042a
							HD	55.843 ± 2.516b	89.623 ± 32.858b	38.328 ± 4.924b
							FD	77.118 ± 2.529b	52.568 ± 3.598b	46.311 ± 2.485b
							EPD	66.282 ± 10.032b	29.473 ± 0.684b	19.569 ± 2.913b
Pentanal‐M	C110623	C5H10O	86.100	699.400	167.833	1.183	HD	101.179 ± 4.643b	87.176 ± 4.600b	122.210 ± 11.282c
							FD	103.399 ± 2.264b	241.621 ± 41.323a	195.133 ± 8.703b
							EPD	236.176 ± 39.906c	279.083 ± 25.319a	291.320 ± 12.316a
Pentanal‐D	C110623	C5H10O	86.100	695.300	166.305	1.421	HD	167.618 ± 9.943b	120.179 ± 12.341c	200.414 ± 70.575c
							FD	179.184 ± 6.410b	745.312 ± 61.878a	466.960 ± 15.895a
							EPD	531.939 ± 57.082a	493.341 ± 38.688b	340.970 ± 33.924b
Butanal	C123728	C4H8O	72.100	604.300	144.527	1.297	Fresh	—	—	—
							HD	219.304 ± 7.613a	170.690 ± 4.342a	273.108 ± 12.875b
							FD	201.645 ± 12.037a	160.890 ± 3.781ab	252.393 ± 7.042b
							EPD	168.472 ± 3.044b	145.991 ± 8.605b	80.674 ± 1.714c
2‐Methylpropanal	C78842	C4H8O	72.100	561.500	134.765	1.283	Fresh	—	—	—
							HD	51.113 ± 1.116b	54.378 ± 9.206b	55.351 ± 8.447b
							FD	16.871 ± 0.898b	36.563 ± 2.071b	24.724 ± 2.135b
							EPD	1045.220 ± 50.830a	745.102 ± 21.815a	363.241 ± 207.906a
Ethyl Acetate	C141786	C4H8O2	88.100	597.800	143.060	1.345	Fresh	5269.666 ± 111.545c	9895.340 ± 941.382b	13673.587 ± 760.919a
Ethyl Acetate‐D	C141786	C4H8O2	88.100	597.800	143.060	1.345	HD	1105.741 ± 48.955b	2921.266 ± 343.161a	1147.969 ± 137.042a
							FD	1802.935 ± 56.470a	165.430 ± 9.261b	502.823 ± 8.508b
							EPD	138.212 ± 0.771c	146.442 ± 5.313b	95.167 ± 3.334c
Ethyl Acetate‐M	C141786	C4H8O2	88.100	614.900	146.938	1.098	HD	107.036 ± 2.893b	86.086 ± 9.496b	115.425 ± 6.871a
							FD	88.625 ± 2.847b	90.630 ± 6.361b	116.939 ± 8.399a
							EPD	212.265 ± 20.869a	166.403 ± 4.623a	113.000 ± 23.880a
Isopropyl acetate	C108214	C5H10O2	102.100	671.500	159.873	1.158	Fresh	—	—	—
							HD	103.547 ± 6.433c	121.664 ± 14.263c	141.557 ± 5.811c
							FD	172.459 ± 2.499b	175.007 ± 14.559b	173.304 ± 13.772b
							EPD	160.837 ± 5.363b	251.321 ± 13.622a	321.823 ± 8.545a
Isoamyl acetate‐D	C123922	C7H14O2	130.200	876.800	247.110	1.758	Fresh	260.751 ± 33.598a	238.941 ± 10.264a	212.515 ± 19.355a
Isoamyl acetate‐M	C123922	C7H14O2	130.200	877.400	247.412	1.306	Fresh	181.644 ± 19.223a	146.557 ± 18.430a	170.799 ± 15.220a
Ethyl 2‐methylbutyrate‐M	C7452791	C7H14O2	130.200	842.500	229.607	1.237	Fresh	248.574 ± 68.886c	851.940 ± 29.426a	680.436 ± 32.881b
Ethyl 2‐methylbutyrate‐D	C7452791	C7H14O2	130.200	841.300	228.978	1.657	Fresh	115.362 ± 45.836c	1910.980 ± 260.900b	1493.435 ± 194.234b
Butyl acetate	C123864	C6H12O2	116.200	802.100	209.033	1.625	Fresh	23.085 ± 2.422a	26.544 ± 1.895a	26.286 ± 2.556a
Methyl hexanoate‐D	C106707	C7H14O2	130.200	920.800	278.803	1.679	Fresh	42.624 ± 0.368b	130.556 ± 43.324a	132.223 ± 36.809a
Methyl hexanoate‐M	C106707	C7H14O2	130.200	923.000	280.674	1.286	Fresh	55.562 ± 7.029b	298.960 ± 56.476a	230.399 ± 33.068a
Propyl butanoate	C105668	C7H14O2	130.200	899.900	261.336	1.269	Fresh	119.404 ± 2.736c	300.075 ± 12.057a	222.937 ± 33.068b
Ethyl butyrate	C105544	C6H12O2	116.200	796.800	206.326	1.209	Fresh	457.547 ± 43.620c	945.153 ± 26.519a	794.541 ± 13.700b
Methyl 2‐methylbutanoate‐D	C868575	C6H12O2	116.200	766.800	193.312	1.538	Fresh	122.495 ± 63.186c	1527.172 ± 183.956a	873.451 ± 179.413b
Methyl 2‐methylbutanoate‐M	C868575	C6H12O2	116.200	761.100	191.168	1.190	Fresh	93.791 ± 21.725c	265.933 ± 9.796a	174.342 ± 14.269b
Ethyl propanoate	C105373	C5H10O2	102.100	699.000	167.712	1.458	Fresh	531.401 ± 48.582c	2494.224 ± 91.023a	1885.258 ± 33.632b
Ethyl 2‐methylpropanoate	C97621	C6H12O2	116.200	744.900	185.024	1.566	Fresh	47.902 ± 2.835b	1154.478 ± 165.030a	887.517 ± 465.394a
Butanoic acid methyl ester‐M	C623427	C5H10O2	102.100	710.600	172.064	1.151	Fresh	556.789 ± 44.446a	550.674 ± 18.603a	313.972 ± 11.947b
Butanoic acid methyl ester‐D	C623427	C5H10O2	102.100	713.100	173.042	1.436	Fresh	422.246 ± 50.955c	2807.515 ± 176.993a	1783.520 ± 46.160b
Acetic acid, 2‐methylpropyl ester	C110190	C6H12O2	116.200	768.800	194.054	1.626	Fresh	74.598 ± 11.830a	79.081 ± 4.300a	112.309 ± 63.736a
Methyl 2‐methylpropanoate	C547637	C5H10O2	102.100	672.700	160.131	1.443	Fresh	37.110 ± 1.868c	431.898 ± 58.071b	112.237 ± 62.725a
Methyl‐5‐hepten‐2‐one	C110930	C8H14O	126.200	992.400	338.524	1.180	Fresh	—	—	—
							HD	161.720 ± 28.139a	79.392 ± 10.266b	51.538 ± 4.314b
							FD	97.618 ± 4.133b	109.610 ± 4.756a	85.377 ± 9.856a
							EPD	88.819 ± 2.614b	73.856 ± 3.497b	81.620 ± 5.179a
Dihydro‐2(3 h)‐furanone‐M	C96480	C4H6O2	86.100	915.800	274.610	1.085	Fresh	—	—	—
							HD	466.433 ± 22.343b	310.184 ± 32.149b	265.071 ± 41.447a
							FD	281.409 ± 113.676c	197.158 ± 79.870bc	136.564 ± 45.807b
							EPD	120.802 ± 18.143c	88.919 ± 5.415c	75.987 ± 5.439b
Dihydro‐2(3 h)‐furanone‐D	C96480	C4H6O2	86.100	916.100	274.913	1.303	Fresh	—	—	—
							HD	158.876 ± 25.815a	50.729 ± 14.575a	19.408 ± 2.965a
							FD	78.640 ± 62.228ab	20.806 ± 11.581b	16.392 ± 2.323ab
							EPD	13.750 ± 2.183b	11.985 ± 2.624b	11.678 ± 1.468b
3‐Pentanone	C96220	C5H10O	86.100	685.500	163.062	1.359	Fresh	140.256 ± 20.210b	359.752 ± 45.463b	867.743 ± 134.379a
							HD	136.100 ± 5.515b	174.092 ± 25.241c	107.049 ± 13.497b
							FD	107.553 ± 6.007b	133.061 ± 133.061c	143.493 ± 5.591b
							EPD	290.105 ± 26.200a	200.998 ± 21.216c	127.328 ± 37.827b
3‐Hydroxybutan‐2‐one‐D	C513860	C4H8O2	88.100	713.400	173.141	1.336	Fresh	—	—	—
							HD	1660.588 ± 103.095a	1707.543 ± 118.062b	1445.091 ± 48.785a
							FD	1058.619 ± 80.341b	553.633 ± 239.055c	546.877 ± 134.512b
							EPD	258.743 ± 86.501c	216.956 ± 17.587c	278.486 ± 62.552c
3‐Hydroxybutan‐2‐one‐M	C513860	C4H8O2	88.100	720.400	175.771	1.057	Fresh	—	—	—
							HD	182.956 ± 10.910b	101.661 ± 9.165b	216.890 ± 29.584b
							FD	165.343 ± 1.716b	296.557 ± 24.141a	264.472 ± 23.887b
							EPD	221.789 ± 43.287b	266.880 ± 12.183a	342.234 ± 32.365a
2,3‐Btanedione	C431038	C4H6O2	86.100	591.100	141.532	1.166	Fresh	—	—	—
							HD	209.421 ± 1.822b	201.618 ± 4.590a	213.140 ± 8.174a
							FD	262.306 ± 4.559a	159.317 ± 1.415b	219.902 ± 0.742a
							EPD	42.767 ± 1.820c	87.718 ± 13.591c	114.161 ± 32.532b
2‐Butanone‐M	C78933	C4H8O	72.100	595.600	142.536	1.061	Fresh	—	—	—
							HD	38.917 ± 2.976c	27.103 ± 1.589c	47.195 ± 0.616b
							FD	29.195 ± 0.480c	102.852 ± 2.049b	59.095 ± 4.995b
							EPD	105.225 ± 6.622b	132.290 ± 8.308a	130.653 ± 10.382a
2‐Butanone‐D	C78933	C4H8O	72.100	587.000	140.586	1.250	Fresh	—	—	—
							HD	138.505 ± 3.334b	182.666 ± 24.761b	171.716 ± 22.662b
							FD	113.643 ± 1.826c	244.982 ± 3.740a	227.442 ± 7.756a
							EPD	419.369 ± 12.263a	246.159 ± 9.142a	141.547 ± 27.831b
Dimethyl sulfide	C75183	C2H6S	62.100	526.300	126.736	0.958	Fresh	—	—	—
							HD	28.981 ± 4.049b	12.274 ± 0.475b	24.570 ± 1.865b
							FD	14.898 ± 2.534b	26.440 ± 2.097b	19.640 ± 0.575b
							EPD	403.939 ± 36.280a	184.477 ± 24.127a	296.198 ± 67.243a
2‐Methyl‐1‐propanol	C78831	C4H10O	74.100	638.400	152.320	1.173	Fresh	—	—	—
							HD	58.862 ± 1.321b	31.626 ± 3.268b	64.053 ± 3.883b
							FD	40.869 ± 1.044c	35.515 ± 3.225b	45.593 ± 2.461c
							EPD	119.049 ± 2.397a	129.794 ± 3.450a	146.803 ± 2.874a

Molecular weight (MW); Retention index (RI); Retention time (RT); Drift time (DT); The letters represent the significant difference (*p* < 0.05) between VOCS of figs after different drying treatments.

## 4 DISCUSSION

Volatile compounds are one of the important factors affecting the aroma of figs. However, different regions, cultivars, and drying treatments can affect the content of volatile compounds. Previous studies have reported that fig substances from the Mediterranean coast were mostly dominated by ketones, alcohols, and aldehydes (Sertkaya et al., 2021). However, our study found that esters were the main volatile compounds in the peel and pulp of fig samples in China by GC‐IMS. Esters, important volatiles in most fruits, are fatty acids synthesized through enzymatic metabolism and contribute to the formation of fig flavor (Sertkaya et al., 2021). In this study, isopropyl acetate, ethyl butyrate, and ethyl propanoate were unique to Bojihong in Shandong, while ethyl 2‐methylbutyrate, propyl butanoate, methyl 2‐methylbutanoate, and ethyl 2‐methylpropanoate were unique to Bojihong peels of Guangzhou. Sadiye et al. (Gozlekci et al., 2011) pointed out that no esters were detected in the peels of two cultivars of Karabakunya and Sultan Selim in Turkey. The two cultivars of Branswick and Banane in Shandong were mostly esters, including methyl 2‐methylbutanoate‐M, butanoic acid methyl ester (M and D), ethyl propanoate, ethyl butyrate, ethyl 2‐methylbutyrate (M and D), and ethyl acetate. In addition, methyl 2‐methylpropanoate, ethyl 2‐methylpropanoate, methyl hexanoate, and methyl 2‐methylbutanoate‐D were detected in the pulps of the two cultivars, while Bojihong was different from the two. Mujić et al. (2014)) found ethyl acetate to be the ester with high content in Croatian dried figs and also found esters such as ethyl ester, methyl salicylate, and 1‐butanol‐3‐methyl acetate, which were not found in figs from China.

Aldehydes are considered to be an important class of volatiles affecting fruit volatiles, which are more likely to be formed through the oxidation of fatty acids or the degradation of amino acids. Benzaldehyde is formed by the catalytic oxidation of benzyl alcohol by dehydrogenase. It was identified as the main compound of figs in Portugal and was also detected in fig samples from Shandong Province. Moreover, furfural, hexanal, and (E)‐2‐hexenal were detected in fig samples from Shandong Province. While in the Bojihong and two other cultivar samples from Guangdong and Sichuan, the aldehyde content was lower with 2‐methylbutanal and 3‐methylbutanal, and these compounds were also reported before (Oliveira et al., 2010).

Drying is an important step in the postprocessing of figs, and the aroma compounds change according to the drying method and temperature changes. Aldehydes are the main aroma compounds in dried figs, and most aldehydes are produced by the cleavage of unsaturated fatty acids through the Maillard reaction. Linear aldehydes (pentanal and butanal) may be derived from the oxidation of linoleic acid (Yang et al., 2021; Zhang et al., 2019); the newly formed 3‐methylbutanal and 2‐methylpropanal are generated by Strecker degradation of leucine and valine, respectively, during the Maillard reaction (Pu et al., 2019). High drying temperatures and long drying times can lead to the loss of esters, resulting in the formation of more alcohols (Guo et al., 2018).

Ketones were confirmed to be the main volatiles of figs after drying (Russo et al., 2017). Higher concentrations were detected in dried treated samples, such as methyl‐5‐hepten‐2‐one, dihydro‐2(3 h)‐furanone (M and D), and 2,3‐butanedione, which increased in content and were probably produced due to oxidation, enzymes, and microbial fermentation.

When the same species of figs were dried in various ways, there were differences in volatile compounds. After vacuum expansion drying treatment, aroma compounds such as 2‐methylpropanal, 2‐butanone, butanal, dimethyl sulfide, 3‐methylbutanal, and 3‐pentanone had great changes. As an effective and promising drying method, explosion puffing drying is applied to many kinds of fruits and vegetables. For example, apples that were pretreated by freezing explosion puffing drying had strong DPPH (1,1‐diphenyl‐2‐picrylhy drazyl free radical – for in vitro antioxidant evaluation), hydroxyl radical, and FRAP (ferric ion reducing antioxidant power – for in vitro antioxidant evaluation) scavenging ability, and the aroma compounds also changed significantly (Feng et al., 2021). Furthermore, vacuum‐expanded and dried yam slices have higher hardness and brittleness, and the volatile components of the product were changed (Gao et al., 2021).

The above results revealed the differences in volatile compounds of figs from three different regions of Shandong, Sichuan, and Guangdong; the differences between Bojihong, Branswick, and Banane; and the differences and trends of Bojihong by hot air drying, freeze‐drying, and explosion puffing drying. It provides a theoretical reference for the development of fig by‐products.

## 5 CONCLUSION

In this paper, the GC‐IMS technique was used to analyze the volatile compounds of the three regions and cultivars, as well as the drying treatments of the figs. Twenty‐seven volatile compounds were detected in the three regions, including 14 esters, 9 aldehydes, 2 ketones, 1 alcohol, and 1 furan. PCA analysis could clearly distinguish the three, and Bojihong from Shandong was mainly hexanal, (E)‐2‐hexenal, benzaldehyde, and furfural. The main volatile compounds of Bojihong from Sichuan and Guangdong were esters such as ethyl acetate, butyl acetate, isoamyl acetate, ethyl butyrate, ethyl propanoate, and acetic acid, 2‐methylpropyl ester, as well as 3‐hydroxybutan‐2‐one and other ketones, and 2‐methylbutan‐1‐ol and other alcohols. Twenty‐eight volatiles were detected in the three cultivars, and PCA results showed that methyl 2‐methylpropanoate, ethyl 2‐methylpropanoate, methyl hexanoate, and methyl 2‐methylbutanoate‐D were specific to Banane and Branswick, compared to Bojihong. Twenty‐nine volatile compounds were detected in figs after treatment with different drying methods. The volatile compounds of the identical cultivars of figs were significantly different after different drying treatments. And there were substantial changes in volatile compounds after drying treatment. This initial progress provides a theoretical basis for further studies on the factors influencing the different discrepancies among figs.

## AUTHOR CONTRIBUTIONS

**Xinyu Liu:** Data curation (equal); formal analysis (equal); writing – original draft (equal). **Rui Sun:** Conceptualization (equal); project administration (equal); supervision (equal); writing – review and editing (equal). **Qiu Wu:** Methodology (equal); resources (equal); visualization (equal); writing – original draft (equal). **Ming Jia:** Validation (equal); visualization (equal); writing – original draft (equal). **Tingjuan Yu:** Validation (equal); visualization (equal); writing – original draft (equal). **Yanling Han:** Formal analysis (equal); visualization (equal); writing – original draft (equal). **Mingguan Yang:** Resources (equal); writing – review and editing (equal). **Lei Sun:** Supervision (equal); writing – review and editing (equal).

## CONFLICT OF INTEREST STATEMENT

The authors declare that they do not have any conflict of interest.

## Data Availability

The authors confirm that the data supporting the findings of this study are available within the article.

## References

[fsn33843-bib-0001] Abdolinejad, R. , Shekafandeh, A. , & Jowkar, A. (2021). In vitro tetraploidy induction creates enhancements in morphological, physiological and phytochemical characteristics in the fig tree (*Ficus Carica* L.). Plant Physiology and Biochemistry, 166, 191–202. 10.1016/j.plaphy.2021.05.047 34118682

[fsn33843-bib-0002] Adiletta, G. , Zampella, L. , Coletta, C. , & Petriccione, M. J. A. (2019). Chitosan coating to preserve the qualitative traits and improve antioxidant system in fresh figs (*Ficus carica* L.). Agriculture, 9(4), 84. 10.3390/agriculture9040084

[fsn33843-bib-0003] Ammar, A. , Aissa, I. B. , Gouiaa, M. , & Mars, M. (2022). Fig (*Ficus carica* L.) vulnerability to climate change: Combined effects of water stress and high temperature on ecophysiological behaviour of different cultivars. South African Journal of Botany, 147, 482–492. 10.1016/j.sajb.2022.02.014

[fsn33843-bib-0004] Arvaniti, O. S. , Samaras, Y. , Gatidou, G. , Thomaidis, N. S. , & Stasinakis, A. S. (2019). Review on fresh and dried figs: Chemical analysis and occurrence of phytochemical compounds, antioxidant capacity and health effects. Food Research International, 119, 244–267. 10.1016/j.foodres.2019.01.055 30884655

[fsn33843-bib-0005] Backes, E. , Pereira, C. , Barros, L. , Prieto, M. A. , Genena, A. K. , Barreiro, M. F. , & Ferreira, I. (2018). Recovery of bioactive anthocyanin pigments from *Ficus carica* L. peel by heat, microwave, and ultrasound based extraction techniques. Food Research International, 113, 197–209. 10.1016/j.foodres.2018.07.016 30195514

[fsn33843-bib-0006] Beaulieu, J. C. , & Stein‐Chisholm, R. E. (2016). HS‐GC‐MS volatile compounds recovered in freshly pressed 'Wonderful' cultivar and commercial pomegranate juices. Food Chemistry, 190, 643–656. 10.1016/j.foodchem.2015.06.005 26213022

[fsn33843-bib-0007] Cagliero, C. , Bicchi, C. , Cordero, C. , Rubiolo, P. , Sgorbini, B. , & Liberto, E. (2012). Fast headspace‐enantioselective GC–mass spectrometric‐multivariate statistical method for routine authentication of flavoured fruit foods. Food Chemistry, 132(2), 1071–1079. 10.1016/j.foodchem.2011.10.106

[fsn33843-bib-0008] Castell, A. , Arroyo‐Manzanares, N. , de Dios Hernandez, J. , Guillen, I. , Vizcaino, P. , Lopez‐Garcia, I. , Hernandez‐Cordoba, M. , & Vinas, P. (2022). Ion mobility spectrometry as an emerging tool for characterization of the volatile profile and identification of microbial growth in pomegranate juice. Microchemical Journal, 174, 107099. 10.1016/j.microc.2021.107099

[fsn33843-bib-0009] Chen, Q. , Li, Z. , Bi, J. , Zhou, L. , Yi, J. , & Wu, X. (2017). Effect of hybrid drying methods on physicochemical, nutritional and antioxidant properties of dried black mulberry. LWT, 80, 178–184. 10.1016/j.lwt.2017.02.017

[fsn33843-bib-0010] Cuevas, F. J. , Moreno‐Rojas, J. M. , & Ruiz‐Moreno, M. J. (2017). Assessing a traceability technique in fresh oranges (Citrus sinensis L. Osbeck) with an HS‐SPME‐GC‐MS method. Towards a volatile characterisation of organic oranges. Food Chemistry, 221, 1930–1938. 10.1016/j.foodchem.2016.11.156 27979182

[fsn33843-bib-0011] Essa, M. M. , Subash, S. , Akbar, M. , Al‐Adawi, S. , & Guillemin, G. J. (2015). Long‐term dietary supplementation of pomegranates, figs and dates alleviate neuroinflammation in a transgenic mouse model of Alzheimer's disease. PLoS One, 10(3), e0120964. 10.1371/journal.pone.0120964 25807081 PMC4373715

[fsn33843-bib-0012] Fella, P. , Kaikiti, K. , Stylianou, M. , & Agapiou, A. (2022). HS‐SPME‐GC/MS analysis for revealing Carob's ripening. Metabolites, 12(7), 656. 10.3390/metabo12070656 35888780 PMC9320592

[fsn33843-bib-0013] Fella, P. , Stylianou, M. , & Agapiou, A. (2023). A green sorptive extraction method (HiSorb‐TD‐GC‐MS) for determining the extra virgin olive oil (EVOO). Aroma Profile, 95(5), 595–610. 10.1515/pac-2023-0202

[fsn33843-bib-0014] Feng, L. , Xu, Y. , Xiao, Y. , Song, J. , Li, D. , Zhang, Z. , Liu, C. , Jiang, N. , Zhang, M. , & Zhou, C. (2021). Effects of pre‐drying treatments combined with explosion puffing drying on the physicochemical properties, antioxidant activities and flavor characteristics of apples. Food Chemistry, 338, 128015. 10.1016/j.foodchem.2020.128015 32932085

[fsn33843-bib-0015] Gao, Q. , Chen, J.‐N. , Zhang, J.‐C. , Liu, C.‐J. , Li, D.‐J. , Liu, C.‐Q. , Tanokura, M. , & Xue, Y.‐L. (2021). Comparison of explosion puffing drying with other methods on the physicochemical properties and volatiles of yam (*Dioscorea opposita thunb*.) chips through multivariate analysis. Drying Technology, 40(7), 1405–1420. 10.1080/07373937.2020.1870488

[fsn33843-bib-0016] Gharibzahedi, S. M. T. , Smith, B. , & Guo, Y. (2019). Ultrasound‐microwave assisted extraction of pectin from fig (*Ficus carica* L.) skin: Optimization, characterization and bioactivity. Carbohydrate Polymers, 222, 114992. 10.1016/j.carbpol.2019.114992 31320048

[fsn33843-bib-0017] Gozlekci, S. , Kafkas, E. , & Ercisli, S. J. N. B. H. A. C.‐N. (2011). Volatile compounds determined by HS/GC‐MS technique in Peel and pulp of fig (*Ficus carica* L.). Cultivars Grown in Mediterranean Region of Turkey, 39(2), 105–108.

[fsn33843-bib-0018] Guo, Y. , Chen, D. , Dong, Y. , Ju, H. , Wu, C. , & Lin, S. (2018). Characteristic volatiles fingerprints and changes of volatile compounds in fresh and dried Tricholoma matsutake singer by HS‐GC‐IMS and HS‐SPME‐GC‐MS. Journal of Chromatography. B, Analytical Technologies in the Biomedical and Life Sciences, 1099, 46–55. 10.1016/j.jchromb.2018.09.011 30241073

[fsn33843-bib-0019] Hearn, L. , Cole, R. , Spadafora, N. D. , & Szafnauer, R. J. A. I. S. P. (2022). Volatile and semi‐volatile compounds in flavoured hard seltzer beverages: Comparison of high‐capacity sorptive extraction (HiSorb). Methods, 3, 100032.

[fsn33843-bib-0020] Hernández‐Mesa, M. , Ropartz, D. , García‐Campaña, A. M. , Rogniaux, H. , Dervilly‐Pinel, G. , & Le Bizec, B. J. M. (2019). Ion mobility spectrometry in food analysis: Principles, current applications and future trends. Molecules, 24(15), 2706. 10.3390/molecules24152706 31349571 PMC6696101

[fsn33843-bib-0021] Leng, P. , Hu, H.‐W. , Cui, A.‐H. , Tang, H.‐J. , & Liu, Y.‐G. (2021). HS‐GC‐IMS with PCA to analyze volatile flavor compounds of honey peach packaged with different preservation methods during storage. LWT, 149, 111963. 10.1016/j.lwt.2021.111963

[fsn33843-bib-0022] Liu, Y.‐J. , Gong, X. , Jing, W. , Lin, L.‐J. , Zhou, W. , He, J.‐N. , & Li, J.‐H. (2021). Fast discrimination of avocado oil for different extracted methods using headspace‐gas chromatography‐ion mobility spectroscopy with PCA based on volatile organic compounds. Open Chemistry, 19(1), 367–376. 10.1515/chem-2020-0125

[fsn33843-bib-0023] Mirheidari, F. , Khadivi, A. , Moradi, Y. , & Paryan, S. (2020). Phenotypic variability of naturally grown edible fig (*Ficus carica* L.) and caprifig (*Ficus carica var. caprificus Risso*) accessions. Scientia Horticulturae, 267, 109320. 10.1016/j.scienta.2020.109320

[fsn33843-bib-0024] Mujić, I. , Bavcon Kralj, M. , Jokić, S. , Jarni, K. , Jug, T. , & Prgomet, Ž. (2012). Changes in aromatic profile of fresh and dried fig ‐ the role of pre‐treatments in drying process. International Journal of Food Science & Technology, 47(11), 2282–2288. 10.1111/j.1365-2621.2012.03099.x

[fsn33843-bib-0025] Mujić, I. , Bavcon Kralj, M. , Jokić, S. , Jug, T. , Subarić, D. , Vidović, S. , Zivković, J. , & Jarni, K. (2014). Characterisation of volatiles in dried white varieties figs (*Ficus carica* L.). Journal of Food Science and Technology, 51(9), 1837–1846. 10.1007/s13197-012-0740-x 25190838 PMC4152540

[fsn33843-bib-0026] Oliveira, A. P. , Silva, L. R. , de Pinho, P. G. , Gil‐Izquierdo, A. , Valentão, P. , Silva, B. M. , Pereira, J. A. , & Andrade, P. B. J. F. C. (2010). Volatile profiling of Ficus carica varieties by HS‐SPME and GC–IT‐MS. Food Chemistry, 123(2), 548–557. 10.1016/j.foodchem.2010.04.064

[fsn33843-bib-0027] Paolucci, M. , Di Stasio, M. , Sorrentino, A. , La Cara, F. , & Volpe, M. G. (2020). Active edible polysaccharide‐based coating for preservation of fresh figs (*Ficus carica* L.). Food, 9(12), 1793. 10.3390/foods9121793 PMC7761759 33287134

[fsn33843-bib-0028] Pico, J. , Gerbrandt, E. M. , & Castellarin, S. D. (2022). Optimization and validation of a SPME‐GC/MS method for the determination of volatile compounds, including enantiomeric analysis, in northern highbush blueberries (*Vaccinium corymbosum* L.). Food Chemistry, 368, 130812. 10.1016/j.foodchem.2021.130812 34419800

[fsn33843-bib-0029] Pu, D. , Zhang, H. , Zhang, Y. , Sun, B. , Ren, F. , Chen, H. , & He, J. (2019). Characterization of the aroma release and perception of white bread during oral processing by gas chromatography‐ion mobility spectrometry and temporal dominance of sensations analysis. Food Research International, 123, 612–622. 10.1016/j.foodres.2019.05.016 31285010

[fsn33843-bib-0030] Russo, F. , Caporaso, N. , Paduano, A. , & Sacchi, R. J. I. J. O. F. P. (2017). Characterisation of volatile compounds in Cilento (Italy) figs (*Ficus carica* L.) cv. Dottato as affected by the drying process. International Journal of Food Properties, 20(sup2), 1366–1376. 10.1080/10942912.2017.1344991

[fsn33843-bib-0031] Sertkaya, M. , Guclu, G. , Buyukkurt, O. K. , Kelebek, H. , & Selli, S. J. F. A. M. (2021). GC‐MS‐olfactometric screening of potent aroma compounds in pulps and peels of two popular Turkish fig (*Ficus carica* L.) cultivars by application of aroma extract dilution analysis. Food Analytical Methods, 14(11), 2357–2366. 10.1007/s12161-021-02057-6

[fsn33843-bib-0032] Slatnar, A. , Klancar, U. , Stampar, F. , & Veberic, R. (2011). Effect of drying of figs (*Ficus carica* L.) on the contents of sugars, organic acids, and phenolic compounds. Journal of Agricultural and Food Chemistry, 59(21), 11696–11702. 10.1021/jf202707y 21958361

[fsn33843-bib-0033] Vallejo, F. , Marín, J. G. , & Tomás‐Barberán, F. A. (2012). Phenolic compound content of fresh and dried figs (*Ficus carica* L.). Food Chemistry, 130(3), 485–492. 10.1016/j.foodchem.2011.07.032

[fsn33843-bib-0034] Villalobos, M. C. , Serradilla, M. J. , Martín, A. , Aranda, E. , López‐Corrales, M. , & Córdoba, M. G. (2018). Influence of modified atmosphere packaging (MAP) on aroma quality of figs (*Ficus carica* L.). Postharvest Biology and Technology, 136, 145–151. 10.1016/j.postharvbio.2017.11.001

[fsn33843-bib-0035] Xiao, Z. , Niu, M. , & Niu, Y. (2022). Comparative study on volatile compounds and taste components of different durian cultivars based on GC‐MS, UHPLC, HPAEC‐PAD, E‐tongue and E‐nose. Molecules, 27(4), 1264. 10.3390/molecules27041264 35209052 PMC8880792

[fsn33843-bib-0036] Yang, F. , Liu, Y. , Wang, B. , Song, H. , & Zou, T. (2021). Screening of the volatile compounds in fresh and thermally treated watermelon juice via headspace‐gas chromatography‐ion mobility spectrometry and comprehensive two‐dimensional gas chromatography‐olfactory‐mass spectrometry analysis. LWT, 137, 110478. 10.1016/j.lwt.2020.110478

[fsn33843-bib-0037] Yu, L. , Meng, Y. , Wang, Z.‐L. , Cao, L. , Liu, C. , Gao, M.‐Z. , Zhao, C. J. , & Fu, Y.‐J. (2020). Sustainable and efficient surfactant‐based microwave‐assisted extraction of target polyphenols and furanocoumarins from fig (*Ficus carica* L.) leaves. Journal of Molecular Liquids, 318, 114196. 10.1016/j.molliq.2020.114196

[fsn33843-bib-0038] Zhang, J. , Cao, J. , Pei, Z. , Wei, P. , Xiang, D. , Cao, X. , Shen, X. , & Li, C. (2019). Volatile flavour components and the mechanisms underlying their production in golden pompano (*Trachinotus blochii*) fillets subjected to different drying methods: A comparative study using an electronic nose, an electronic tongue and SDE‐GC‐MS. Food Research International, 123, 217–225. 10.1016/j.foodres.2019.04.069 31284971

[fsn33843-bib-0039] Zidi, K. , Kati, D. E. , Bachir‐Bey, M. , Genva, M. , & Fauconnier, M. L. (2021). Comparative study of fig volatile compounds using headspace solid‐phase microextraction‐gas chromatography/mass spectrometry: Effects of cultivars and ripening stages. Frontiers in Plant Science, 12, 667809. 10.3389/fpls.2021.667809 34276728 PMC8283200

